# Scenario of Firearm Injuries in Saudi Arabia: A Comparative Review

**DOI:** 10.7759/cureus.54946

**Published:** 2024-02-26

**Authors:** Usama Bin Ghaffar

**Affiliations:** 1 Department of Basic Sciences, Majmaah University, College of Medicine, Al Majma'ah, SAU

**Keywords:** wound, management, violence, saudi arabia, firearm injury

## Abstract

The global concern regarding the involvement of firearms in acts of violence, criminal activities, and terrorism has escalated, emerging as a leading cause of mortality and morbidity worldwide. The Kingdom of Saudi Arabia (KSA), with its rich cultural history, presents a unique backdrop characterized by diversity and distinctiveness. Recognizing the significance of addressing this issue within diverse demographics, we have undertaken a study to establish a database of firearm injury cases. This review is rooted in analyzing all published articles on firearm injuries in the KSA over the past three decades. The literature encompasses a substantial number of studies with diverse objectives, covering a range of parameters, including the types of cases (homicide, suicide, and accidental), the weaponry involved, the location of gunshot wounds, victim demographics (such as age), the timing of gunshot-related fatalities, and more. Studies consistently indicate that the lower extremities are the most frequently affected body regions, followed by the upper extremities and chest. This information will be a scientific, evidence-based resource to educate the public about firearm-related risks. In addition, it will aid in planning appropriate interventions and formulating stricter laws to regulate the issuance of firearm licenses and impose severe penalties for carrying firearms in public spaces. The ultimate goal is to prevent the loss of life and mitigate the lifelong disabilities resulting from firearm-related incidents.

## Introduction and background

Firearm injury represents a significant threat to the health and safety of all individuals, with a higher prevalence both in developing and developed countries [1]. Each year, gun-related injuries claim the lives of hundreds of thousands of people [2]. Violence involving guns in the United States (US) is a vigorously debated political topic. In addition to gang violence, which frequently affects minors or young adults, violence involving firearms is particularly prevalent in impoverished urban areas. In comparison to deaths from motor vehicle accidents, the danger of firearm-related injuries is higher in the US. The rates of firearm-related deaths, however, are lower in European nations [3]. Gun-related injuries superseded car accidents, cancerous neoplasms, drug overdoses, and poisonings to become the top cause of death in the United States for individuals aged one to 19 in 2020 (83% increase since 2013) [4]. Over half of all firearm-related injury deaths in 2016 occurred in a few high-prevalence countries: Brazil, the US, Colombia, Mexico, Venezuela, and Guatemala [5].

The widespread, uncontrolled use of firearms, especially in the past 10 years, is a cause for increasing concern. Some have compared the spread of small arms and light weapons (SALWs) in underdeveloped countries to the spread of cancer. It causes harm, death, and anarchy while upending social, political, and economic structures [6,7,8]. Many individuals do not believe that an air gun is dangerous. Although it may not appear hazardous at first, it can be lethal. Regretfully, people regard these weapons as toys for kids.

Consequently, the frequency of air pistol mishaps is rising. Air gun injuries are typically lethal unless they hit the head, with children being the most common victims [9]. Firearms are frequently associated with pride in many countries, mainly in rural areas. The number of gun-related deaths has reached an all-time high in some countries due to easy access to locally produced firearms, as well as an increase in societal stress, adultery, familial troubles, interpersonal disputes, and a breakdown of moral and familial ties [10].

Firearms were a factor in 0.3% of deaths in Turkey in 2008, and like other nations, this is currently a significant public health issue [11]. According to studies conducted by the University of Sydney's International Gun Policy Facts research team, the Saudi Arabian population is estimated to own 35.0 private firearms for every 100 people. The Kingdom of Saudi Arabia (KSA) is ranked 16th out of 178 countries in a 2007 comparison of the number of privately owned firearms in the world (www.GunPolicy.org, The University of Sydney International Research on Gun Policy Facts). In addition, Waiselfiz reported that there are 82/100,000 gun-related deaths in the KSA [12].

The KSA's legislation mandates that only members of the general public may purchase guns. Unfortunately, the KSA does not currently have a centralized registry of firearm-related fatalities and injuries. Efforts to record and audit firearm-related injury data are required to rule out disparities between data gathered from the health system and police records. The medical literature does contain a limited number of reports regarding firearm-related injuries and deaths that have occurred in certain parts of the KSA. The majority of the KSA's civilian doctors will hardly ever get the opportunity to treat gun-related injuries. These injuries are primarily treated by military surgical services, local hospitals, and trauma centers [13].

The objective of this review was to critically examine earlier-published research articles from the previous 30 years in the KSA to determine the demographic figures, distribution of injuries, new developments, and essential strategies for preventing firearm injuries. Concerning reducing the overall frequency, severity, and resulting injuries from firearms in the KSA, this analysis seeks to offer a more insightful and better understanding.

## Review

Methodology

Between August 2023 and October 2023, the keywords "firearm injuries," "Saudi Arabia," and "gunshot injuries" were searched in PubMed, Google Scholar, the Saudi Digital Library (SDL), UpToDate, Saudi Medical Literature, and Science Direct. In addition, the websites of the Saudi Ministry of Interior (MOI) and the World Health Organization were the primary sources for gathering data for this review paper. Using all of the articles, news, and bulletins from the past 30 years, both inside and outside the KSA, a database for analyzing the research's goal was constructed. This review only included articles published in English and withdrew all papers written in other languages.

**Figure 1:**
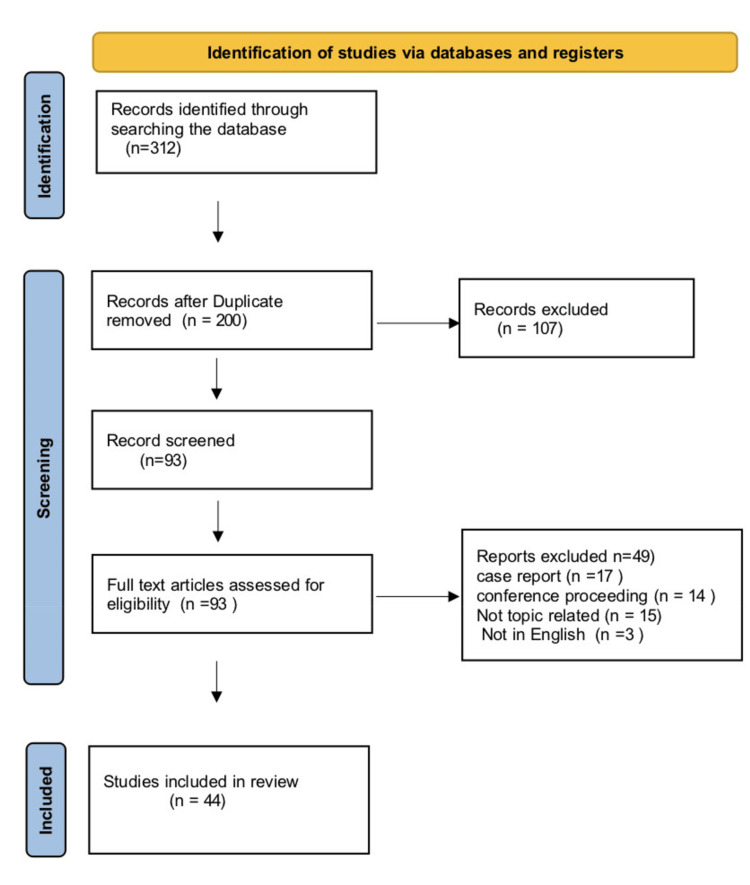
Inclusion and exclusion criteria

To determine the outcome measures, the author independently examined 44 research papers deemed relevant to the issue. The evaluation's outcomes for firearm-related injuries included injury or impairment and deaths at the scene of the event or in the hospital. Figure 1 mentions the inclusion and exclusion criteria of this review.

Results and discussion

We evaluated 44 articles from around the world on firearm-related injuries. Five articles from the region were found, with the study focusing primarily on gunshot injuries in the KSA. The studies showed that male youths experienced more injuries than female youths during the review process (Table 1).

**Table 1:** Comparative overview of different studies with findings on firearm injuries in Saudi Arabia

Study authors	Study design	Study location	No. of participants	Age	Sex	Cause	Common site of injury	Type of weapon
Morgan et al., 2019 [13]	CS	King Khalid Hospital, Alkharj	102	20-30 years (43.1%)	Males = 98 (96.1%), females = 4 (3.9%)	Domestic violence (65.7%) or unintentional fire (17.7%)	Lower limb (43.4%), abdomen (19.6%)	Small firearm (64.7%), airgun (35.3%)
Alassiri, 2013 [14]	CS	Aseer Central Hospital	118	Mean age 26.7 ± 11.1	Males = 117 (99.2%), females = 1 (0.8%)	Intentional (80.5%), unintentional (19.5%)	Lower limb (52.5%), upper limb (22%), trunk (17.8%)	Pistol/handgun (85.6%), airgun (14.4%)
Osama et al., 2008 [15]	CS	Forensic Medicine Centre, Dammam	64	16-31 years (40%), 31-45 years (37.5%)	Males = 59 (92.2%), females = 5 (7.8%)	Homicide (86%), suicide (10.9%), accidental (3.1%)	Head (36%), chest (28%)	Handgun (76.5%), rifle (23.5%)
Softah et al., 2002 [16]	CS	Asir Central Hospital, Abha	42		Males = 42 (100%)	Accidental (2.4%)	Lower limb (47.2%), upper limb (22%), chest (13%)	Pistol (81%), airgun (9.5%)
Elfawal and Awad, 1997 [17]	CS	Eastern province, King Faisal University, Dammam	71		Males = 71 (100%)	Homicide (48%), suicide (28%), accidental (24%)	Chest (41%), head (34%)	Handguns

The consistent reporting of a higher number of male fatalities compared to females in firearm-related incidents is evident both in our review and worldwide. Similar findings from studies by Mirza et al. [18], Kumari et al. [10], and Vithalrao et al. [19] are also in support of this trend. Consequently, men face a heightened vulnerability to conflicts, negative behaviors, hatred, and outdoor violence because they are generally assumed to fulfill the responsibility of providing financial support for the family.

Morgan et al. [13] showed that 43.1% of firearm injuries occurred in the age group 20-30 years, of whom 96.1% were males and 3.9% were females. Another study by Alassiri [14] showed a mean age of 26.7 ± 11.1 years. This finding is consistent with most of the studies covering firearm deaths by Kohli et al. [20], Singh et al. [21], Pattowary et al. [22] from India, Fedakar et al. [23] from Turkey, and Hagras et al. [24] from Egypt. Studies carried out by Karger et al. [25] in Germany and Onuminya [26] in Nigeria also revealed that most victims were male in the age group of 20-30 years because the youth of this age group had a high level of aggression; they indulged in more conflicts than the older population. Elderly individuals possess greater wisdom and exhibit self-control; they tend to refrain from engaging in disputes rather than instigating them. Men are more prone to stress and unhappiness in their daily routines due to their frequent presence in work environments characterized by the presence of violence.

Gender-based violence

Men are disproportionately affected since they are the primary breadwinners and have the responsibility to maintain the moral values of the household and address any possible threats that could escalate into violence. On the other hand, women typically limit themselves to engaging in indoor activities and are less likely to engage in fights that could lead to murder. Since young men tend to be more daring and aggressive, there is a global trend toward male dominance. The aggravating factors are romantic relationships and aggression. Older people and children were the least impacted [27].

Results revealed that the Al Kharj region reported 3.13 cases of firearm injuries per 100,000 individuals. The findings from a study conducted in the Abha region of the KSA, as reported by Softah et al. [16], indicate that this figure is around 2.5 times smaller. The variations in the population composition of the nationalities living in these areas may cause the disparities in these rates. A significant proportion of incidents occurring in the Middle East involve juveniles, significantly impacted by sociological and legal conditions that are analogous to those prevalent in Western societies.

Factors related to firearm injuries

In a study conducted in Dammam, Osama et al. [15] found that homicide was the primary cause of firearm injuries at 86%, followed by suicide at 10.9% and unintentional accidents at 3.1%. Following Elfawal and Awad [17], who noted 48% of homicide, 28% of suicide, and 24% of unintentional cases, Alassiri [14] reported similar findings, with homicide being the predominant cause at 80.5%. This pattern of results consistently indicates that homicide constitutes the highest proportion of firearm injuries, followed by suicides and accidents. Azumak et al. [28] reported a comparable outcome in Turkey, where homicides represented 68.3% of firearm fatalities. Amiri et al. [29], in a study in Tehran, found that 60.7% of cases were homicides, 30.3% were suicides, and 4.5% were accidental. Similarly, Solarino et al. [30] in Italy, Khetran et al. [31] in Baluchistan, and Kohli et al. [32] in India all reported that the majority of firearm fatality cases were attributable to homicidal incidents.

Suicide

However, a study on firearm fatalities by Lemaire et al. [33] in the USA discovered that there was a higher use of firearms in suicidal matters. In certain Western nations, fatal injuries caused by firearms are the leading contributors to both suicides and homicides, primarily due to the ease of access and legal acquisition of firearms for self-defense purposes. Another investigation conducted by Thomsen et al. [34] in Denmark and Norton and Langley [35] in New Zealand indicated that suicides constituted the majority of firearm fatalities, accounting for 80% and 75.5%, respectively. The heightened frequency of homicidal deaths might be attributed to the substantial use of unlicensed firearms, often smuggled to commit terrorist acts.

Matzopoulos [36], Meel [37], Rainio and Sajantila [38], and other researchers have reported similar findings regarding these factors and the impact of social, ethnic, and devotional customs and ideologies. In certain Western nations, fatal firearm injuries predominate as the primary cause of suicides and homicides due to the convenient accessibility and lawful acquisition of firearms for self-defense purposes. On the other hand, since wealthy people own most legal guns, homicides account for the majority of cases in nations like India, where illegal and locally made firearms are more affordable for criminal use.

Location of firearm injuries

Examining the location of injuries across various studies revealed consistent patterns. According to Morgan et al. [13], the lower limb was the area most frequently affected, accounting for 43.4% of all cases, and the abdomen came in second with 19.6%. Similarly, Alassiri [16] reported that the lower limb was the most commonly affected region at 52.5%, with the upper limb following at 22%. Osama et al. [26] discovered that the chest (28%), rather than the head (36%), was the most typical location. Husain et al. [39] conducted a study in Peshawar and found that the extremities were the sites most commonly attacked. Turgut et al. [40] in Turkey also reported similar observations. In contrast to the findings of Karlsson et al. [41] in Sweden, who identified the head as the primary target region, this research presents a different perspective.

Khetran et al. [31] in Baluchistan noted that the chest and abdomen were the most common target sites. Azmak et al. [28] in Turkey reported the chest as the most frequent site for wounds. Edirisinghe et al. [42] in Sri Lanka found that the head or chest was the most commonly targeted area. In addition, Das Gupta et al. [43], Potwary [19], Kohli [17], and Vithalrao et al. [19] in India observed that the majority of victims had entry wounds in the chest. Perpetrators often focus on crucial areas, such as the head or chest. Contrarily, people who take their own lives often target sensitive regions, such as the head or mouth. By contrast, unintentional gunshot wounds or threats usually injure less vital areas, such as the upper or lower limbs [21].

Weapons in firearm injuries

Examining the choice of weapons, Morgan et al. [13] documented that small firearms constituted 64.7%, with airguns accounting for 35.3%. Alassiri et al. [16] observed a prevalence of 85.6% for pistols and handguns and 14.4% for airguns. In Osama et al.'s study [26], handguns were identified at 76.5%, while rifles constituted 23.5%. The growing prevalence of pistols and handguns is consistent with the research findings of Fedakar et al. [20], Azmak et al. [28], Meel [37], and Rainio and Sajantila [38]. This trend can be linked to these firearms being readily available for purchase and smuggling. Furthermore, the price and ease of obtaining and carrying pistols contribute to this pattern [44]. Most Saudis would view an air gun more as a toy than a potentially lethal weapon. The lack of knowledge about air guns as weapons, which can occasionally be fatal but can cause serious injuries, is most likely the cause of this population's mindset.

## Conclusions

Firearm injuries pose a significant public health challenge, exerting a profound impact on a nation's overall safety and well-being. Employing a public health approach is crucial for addressing firearm violence and ensuring the safety and health of the population. In comparison to other developed countries, the KSA ranks among the lowest in personal firearm ownership, owing to stringent national gun regulations, distinguishing it from developed regions in Europe and North America.

A detailed analysis of murder statistics from throughout the world reveals that a wide range of behaviors ultimately lead to the same outcome. Family conflicts, unemployment, terrorism, and other societal changes are just a few of the factors that have an impact on these behaviors. Contributing variables in our particular location might be blood feuds and deeply ingrained customs surrounding the possession and usage of firearms.

The maintenance of law enforcement and security stability is paramount to curbing the proliferation of firearm violence. In addition, implementing new restrictions to eliminate illegal firearm sales is crucial. Educational initiatives are essential to enhance youth awareness about the consequences of violence, while efforts to improve economic conditions and combat ignorance can also play a pivotal role. Rigorous arms inspections by law enforcement agencies and the establishment of a surveillance system to control violence may contribute significantly to reducing the burden of firearm-related fatalities.
